# Supplementary material for: p-wave superconductivity in iron-based superconductors
Source: Sci Rep. 2019 Oct 2;9:14245. doi: 10.1038/s41598-019-50687-y (PMC6775168; doi:10.1038/s41598-019-50687-y)
Supplement: Supplementary file 1 — Supplementary Information [file 41598_2019_50687_MOESM1_ESM.pdf]

Supplementary Information

_p_-wave superconductivity in iron-based superconductors

E. F. Talantsev[1,2,*], K. Iida[3,4], T. Ohmura[3], T. Matsumoto[4], W. P. Crump[5,6], N. M. Strickland[5], S. C. Wimbush[5,6] and H. Ikuta[3,4]

[1] M. N. Mikheev Institute of Metal Physics, Ural Branch, Russian Academy of Sciences, 18 S. Kovalevskoy St., Ekaterinburg 620108, Russia

[2] NANOTECH Centre, Ural Federal University, 19 Mira St., Ekaterinburg 620002, Russia

[3] Department of Crystalline Materials Science, Nagoya University, Chikusa-ku, Nagoya 464-8603, Japan

[4] Department of Materials Physics, Nagoya University, Chikusa-ku, Nagoya 464-8603, Japan

[5] Robinson Research Institute, Victoria University of Wellington, 69 Gracefield Road, Lower Hutt 5010, New Zealand

[6] MacDiarmid Institute for Advanced Materials and Nanotechnology, PO Box 33436, Lower Hutt 5046, New Zealand

*Corresponding author: E-mail: evgeny.talantsev@imp.uran.ru

Supplementary Table I. BCS weak-coupling limit values for $2\Delta(0)/k_BT_c$ and for $\Delta C/C$ and low-temperature asymptotes for the superfluid density, $\rho_s(T)$, for *s*-, *d*-, and *p*-wave pairing [1-5]. For hybrid states the power law exponents were deduced by fittings of the calculated curves of Gross-Alltag *et al.* [2] to the given function, where A and B were free fitting parameters of the order of unity; k_B is the Boltzmann constant; $\Delta_m(0)$ is the maximum amplitude of the *k*-dependent *d*-wave gap, $\Delta(\theta) = \Delta_m(0)\cos(2\theta)$; $\varsigma(3) = 1.2020$ is Riemann's zeta function.

**Pairing symmetry and experiment geometry**	$\frac{2\Delta(0)}{k_BT_c}$	$\frac{\Delta C}{C}$	$\rho_s(T)$ low-T asymptote
s-wave	3.53	1.43	$1-2\sqrt{\frac{\pi\Delta(0)}{k_BT}}e^{-\frac{\Delta(0)}{k_B\cdot T}}$
d-wave	4.28	0.995	$1-2\frac{k_B\cdot T}{\Delta_m(0)}$
p-wave; polar **A**⊥***l***	4.92	0.792	$1-\frac{3\pi ln(2)}{2}\left(\frac{k_BT}{\Delta(0)}\right)^1$
p-wave; polar **A**||***l***	4.92	0.792	$1-\frac{27\pi\varsigma(3)}{4}\left(\frac{k_BT}{\Delta(0)}\right)^3$
p-wave; axial **A**⊥***l***	4.06	1.19	$1-\frac{7\pi}{15}\left(\frac{k_BT}{\Delta(0)}\right)^4$
p-wave; axial **A**||***l***	4.06	1.19	$1-\pi^2\left(\frac{k_BT}{\Delta(0)}\right)^2$
p-wave; hybrid **A**⊥***l***	4.22	0.998	$1-A\left(\frac{k_BT}{\Delta(0)}\right)^{1.2}$
p-wave; hybrid **A**||***l***	4.22	0.998	$1-B\left(\frac{k_BT}{\Delta(0)}\right)^{2.6}$

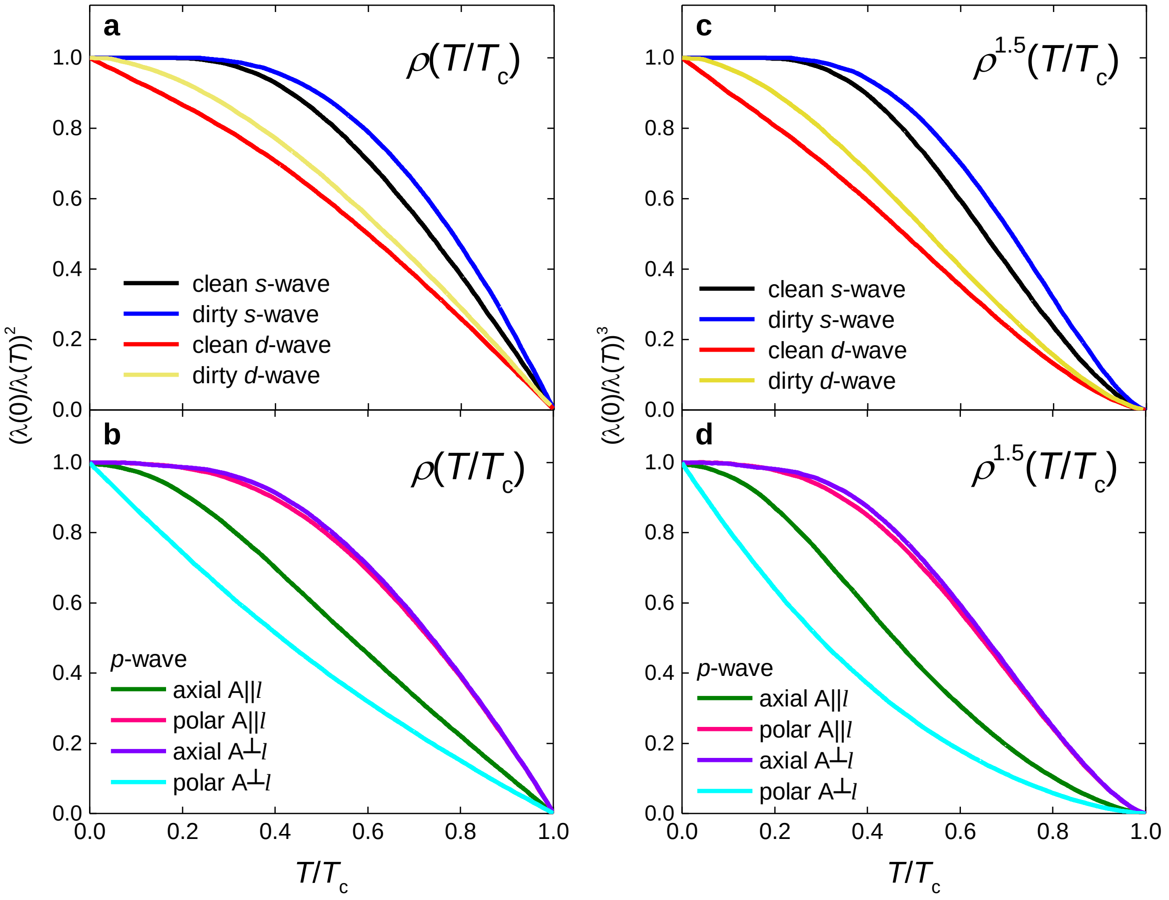

Supplementary Figure 1. Calculated normalized superfluid density $\rho_s(T/T_c)$ for (a) *s*- and *d*-wave superconductors, and (b) *p*-wave superconductors. The same results plotted as $\rho_s^{1.5}(T/T_c)$ for (c) *s*- and *d*-wave superconductors, and (d) *p*-wave superconductors.

Superfluid density measurements of bulk samples

Below we demonstrate that the temperature-dependent superfluid density, $\rho_s(T)$, measured using muon-spin rotation (μSR) spectroscopy for most iron-based superconductors reported in the literature is also consistent with *p*-wave pairing symmetry in these materials.

$(Li_{0.84}Fe_{0.16})OHFe_{0.98}Se$ single crystals. In Fig. S2 we show the experimental data for $\rho_{s,ab}(T)$ (the superfluid density in the *a*-*b* plane) reported by Khasanov *et al.* [6] and a fit using the axial **A⊥*l*** *p*-wave model. There were not enough raw data points near T_c, and thus to increase the accuracy of the derived parameters, we reduced the number of free parameters by fixing T_c to the last experimental data point.

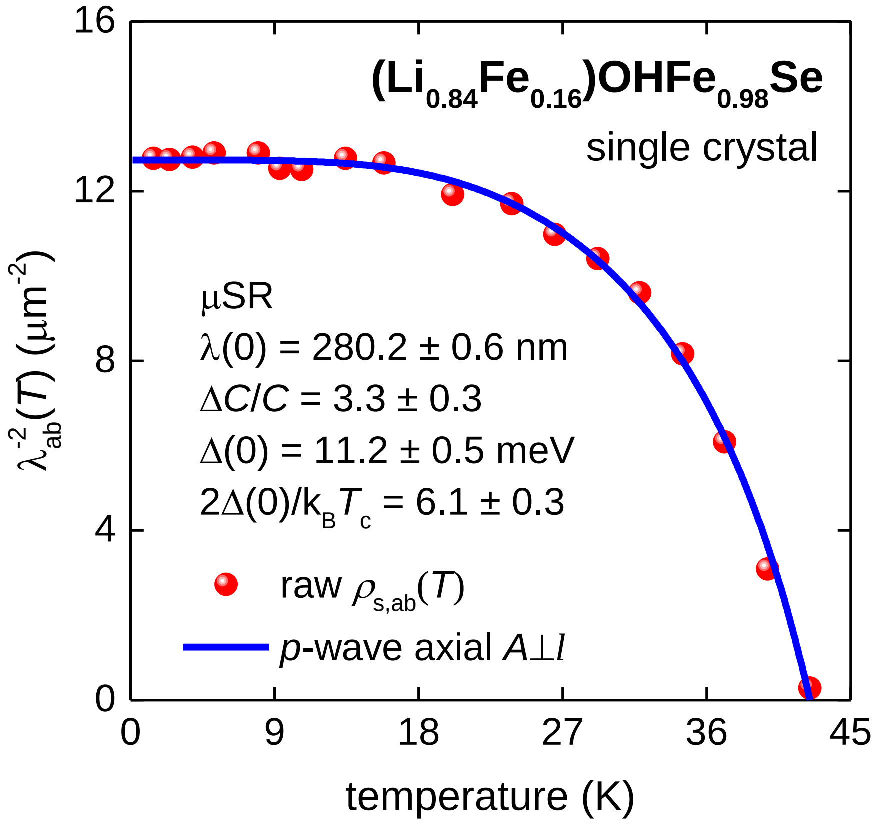

Supplementary Figure 2. BCS fits to the experimental $\rho_{s,ab}(T)$ data for a $(Li_{0.84}Fe_{0.16})OHFe_{0.98}Se$ single crystal [6] assuming a *p*-wave axial **A⊥*l*** model. Derived parameters are: T_c = 42.5 K (fixed), Δ(0) = 11.2 ± 0.5 meV, Δ*C*/*C* = 3.3 ± 0.3, λ(0) = 280.2 ± 0.6 nm, $2\Delta(0)/k_BT_c$ = 6.1 ± 0.3. Fit quality is *R* = 0.9897.

As can be seen, Δ(0) and 2Δ(0)/$k_B T_c$ values deduced from both J_c(sf,T) and μSR data are in excellent agreement with each other, and both indicate moderately strong coupling with *p*-wave gap symmetry in the (Li,Fe)OHFeSe superconductor.

$Rb_{0.77}Fe_{1.61}Se_2$ single crystals. We examine next the μSR measurements of single crystal $Rb_{0.77}Fe_{1.61}Se_2$ reported by Shermadini *et al.* [7]. In Fig. S3 we show the raw $\rho_{s,ab}(T)$ data with a fit using the *p*-wave axial **A⊥*l*** model, where again to increase the accuracy of the deduced parameters we fixed the T_c to the last experimental data point.

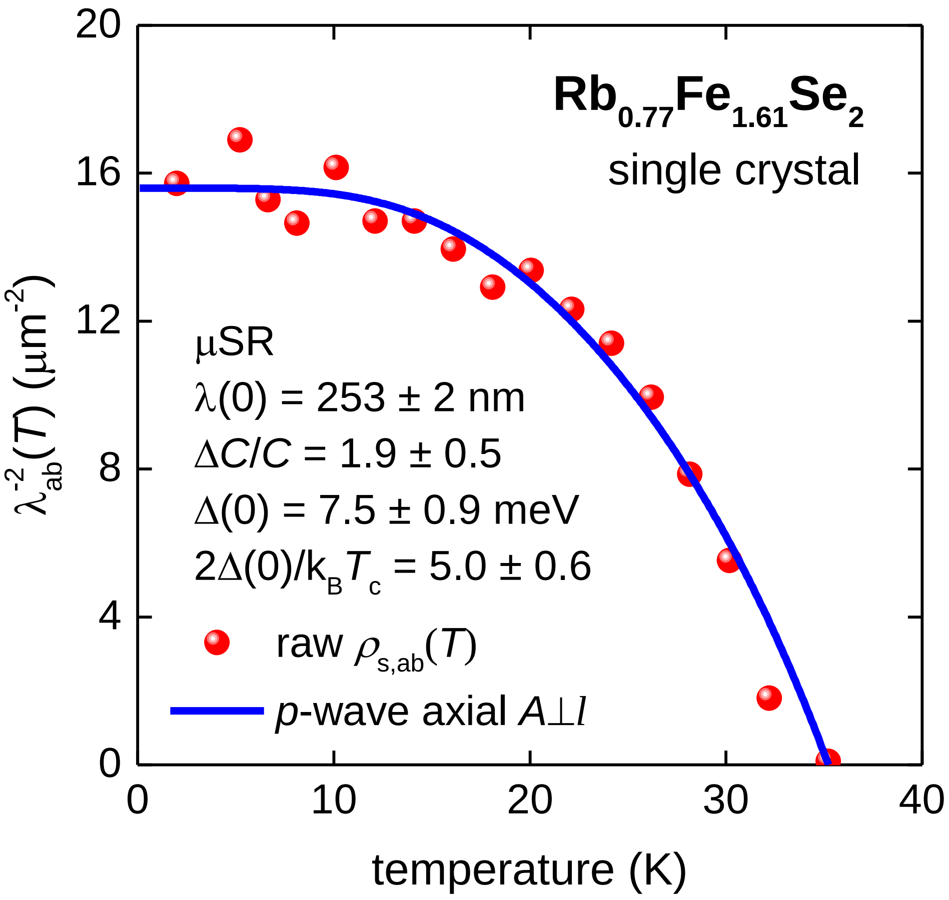

Supplementary Figure 3. BCS fits to the experimental $\rho_{s,ab}(T)$ data for a $Rb_{0.77}Fe_{1.61}Se_2$ sample [7] assuming a *p*-wave axial **A⊥*l*** model. Derived parameters are: T_c = 35.22 K (fixed), Δ(0) = 7.5 ± 0.9 meV, Δ*C*/*C* = 1.9 ± 0.5, λ(0) = 253 ± 2 nm, 2Δ(0)/$k_B T_c$ = 5.0 ± 0.6. Fit quality is R = 0.8764.

We note that Shermadini *et al.* [7] fitted their data to an *s*-wave model and they deduced a very similar value for $\Delta(0) = 7.7$ meV, which can be compared with our value of $\Delta(0) = 7.5 \pm 0.9$ meV. However, the fit to an *s*-wave model has an unavoidable problem which is the value for the ratio $2\Delta(0)/k_BT_c = 5.5$. This is unrealistically large compared with all other known *s*-wave superconductors [8], and especially the weak-coupling limit of BCS theory of 3.53. By way of comparison, Pb which is a strongly-coupled *s*-wave superconductor has $2\Delta(0)/k_BT_c = 4.86$ [9].

Despite the fact that our ratio is essentially the same $2\Delta(0)/k_BT_c = 5.0 \pm 0.6$, we need to stress that the weak-coupling value for this *p*-wave axial $\mathbf{A}\perp\boldsymbol{l}$ case is $2\Delta(0)/k_BT_c = 4.06$, which places $Rb_{0.77}Fe_{1.61}Se_2$ as a moderately strongly coupled superconductor. In addition, the deduced $\Delta C/C = 1.9 \pm 0.5$ is not too far from the weak-coupling limit of $\Delta C/C = 1.2$.

$K_{0.74}Fe_{1.64}Se_2$ single crystals. Shermadini *et al.* [7] also studied in the same paper another iron-based superconductor $K_{0.74}Fe_{1.64}Se_2$. In Fig. S4 we show the raw $\rho_{s,ab}(T)$ data and a fit using the same *p*-wave axial $\mathbf{A}\perp\boldsymbol{l}$ model, where again the T_c was fixed to a rounded value close to the last experimental data point.

As was the case for $Rb_{0.77}Fe_{1.61}Se_2$, Shermadini *et al.* [7] also fitted their data to an *s*-wave model and found $\Delta(0) = 6.3$ meV. In our case we also found this same value ($\Delta(0) = 6.3 \pm 0.4$ meV). However, again for an *s*-wave model the ratio $2\Delta(0)/k_BT_c = 4.7$ is large compared with the majority of other known *s*-wave superconductors [9]. In the *p*-wave case $2\Delta(0)/k_BT_c = 4.5 \pm 0.3$ is in good agreement with a moderately strong coupling pairing strength.

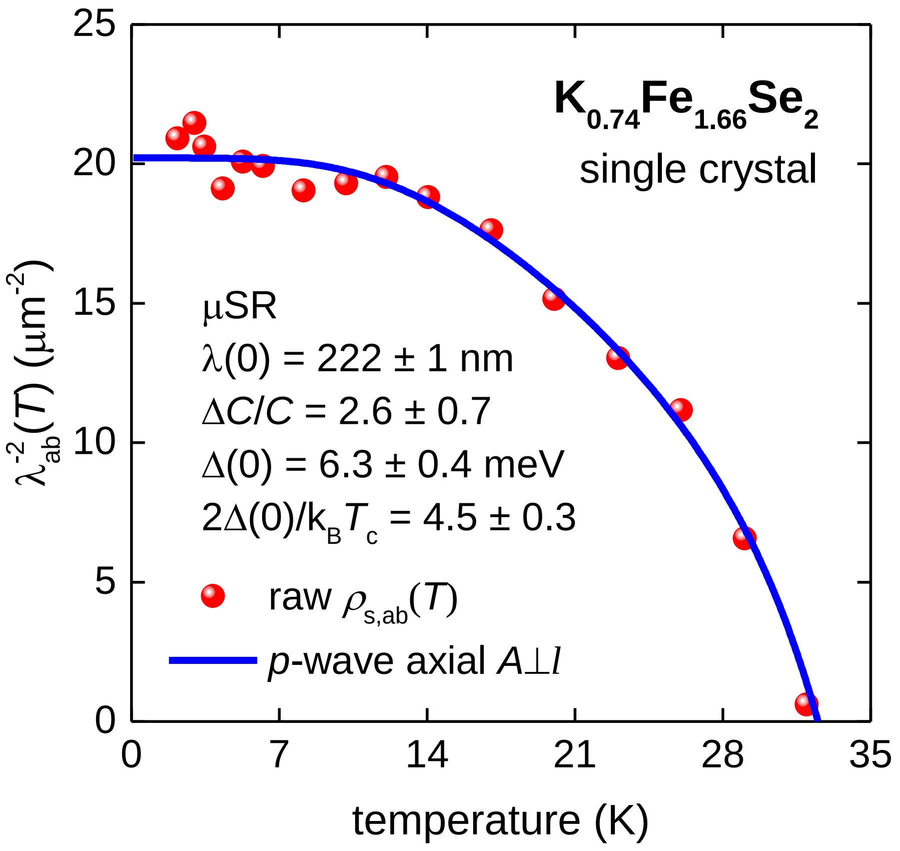

Supplementary Figure 4. BCS fits to the experimental $\rho_{s,ab}(T)$ data for a $K_{0.74}Fe_{1.66}Se_2$ sample [7] assuming a *p*-wave axial **A⊥*l*** model. Derived parameters are: T_c = 32.5 K (fixed), Δ(0) = 6.3 ± 0.4 meV, Δ*C*/*C* = 2.6 ± 0.7, λ(0) = 222 ± 1 nm, $2\Delta(0)/k_BT_c$ = 4.5 ± 0.3. Fit quality is *R* = 0.8981.

Supplementary References

1. Gross., F., *et al.* Anomalous temperature dependence of the magnetic field penetration depth in superconducting UBe_{13}. *Z. Phys. B* **64**, 175-188 (1986).
2. Gross-Alltag, F., Chandrasekhar, B. S., Einzel, D., Hirschfeld, P. J., and Andres, K. London field penetration in heavy fermion superconductors. *Z. Phys. B* **82**, 243-255 (1991).
3. Bardeen, J., Cooper, L. N., and Schrieffer, J. R. Theory of superconductivity. *Phys. Rev.* **108**, 1175-1204 (1957).
4. Hirschfeld, P. J., Korshunov, M. M. and Mazin, I. I. Gap symmetry and structure of Fe-based superconductors. *Rep. Prog. Phys.* **74**, 124508 (2011).
5. Hosono, H. and Kuroki, K. Iron-based superconductors: Current status of materials and pairing mechanism. *Physica C* **514**, 399-422 (2015).
6. Khasanov, R., *et al.* Proximity-induced superconductivity within the insulating $(Li_{0.84}Fe_{0.16})OH$ layers in $(Li_{0.84}Fe_{0.16})OHFe_{0.98}Se$. *Phys. Rev. B* **93**, 224512 (2016).
7. Shermadini, Z., *et al.* Superconducting properties of single-crystalline $A_xFe_{2-y}Se_2$ (A=Rb, K) studied using muon spin spectroscopy. *Phys. Rev. B* **85**, 100501(R) (2012).
8. Poole C. P., Farach H. A., Creswick, R. J., and Prozorov, R. *Superconductivity* (2nd edition, Amsterdam: Elsevier, 2007).
9. Nicol E. J. and Carbotte, J. P. Comparison of pressurized sulfur hydride with conventional superconductors. *Phys. Rev. B* **91**, 220507(R) (2015).
